# Fracture Toughness of Advanced Ceramics at Room Temperature

**DOI:** 10.6028/jres.097.026

**Published:** 1992

**Authors:** George D. Quinn, Jonathan Salem, Isa Bar-on, Kyu Cho, Michael Foley, Ho Fang

**Affiliations:** National Institute of Standards and Technology, Gaithersburg, MD 20899; National Aeronautics and Space Administration, Lewis Research Center, Cleveland, OH 44135; Worcester Polytechnic Institute, Worcester, MA 01609; St. Gobain, Norton Industrial Ceramics Corp., Northboro, MA 01532; Allied-Signal, Garrett Auxiliary Power Division, Phoenix, AZ 85010

**Keywords:** advanced ceramic, alumina, fracture, fracture toughness, indentation, round-robin, silicon nitride, zirconia

## Abstract

This report presents the results obtained by the five U.S. participating laboratories in the Versailles Advanced Materials and Standards (VAMAS) round-robin for fracture toughness of advanced ceramics. Three test methods were used: indentation fracture, indentation strength, and single-edge pre-cracked beam. Two materials were tested: a gas-pressure sintered silicon nitride and a zirconia toughened alumina. Consistent results were obtained with the latter two test methods. Interpretation of fracture toughness in the zirconia alumina composite was complicated by R-curve and environmentally-assisted crack growth phenomena.

## 1. Introduction

The Versailles Advanced Materials and Standards (VAMAS) project is an international collaboration for prestandardization research. The participating countries are Canada, France, Germany, Italy, Japan, the United Kingdom, the United States and the Commission of European Communities. Technical Working Area #3, Ceramics, has the objective of undertaking research on the reliability and reproducibility of test procedures for advanced technical ceramics.

Fracture toughness is an important property of advanced ceramics and is one measure of brittleness. The Japan Fine Ceramics Center (JFCC) in 1988 organized a VAMAS round-robin to evaluate fracture toughness by three methods on two advanced ceramics. All testing was to be performed at room temperature. This round-robin was designated the ’89 Fracture Toughness Round-Robin Test (RRT) by the JFCC. Twenty-three laboratories agreed to participate, including six in the United States.

**Fig. 1:**
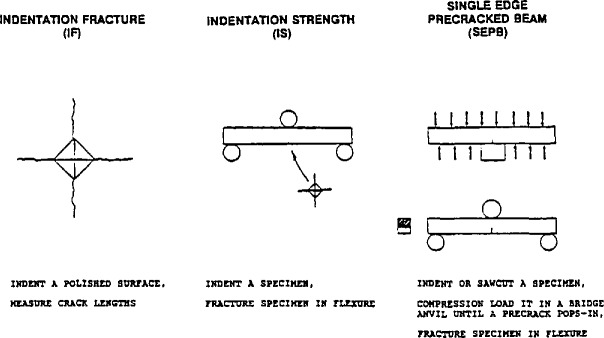
The test methods used for the VAMAS Round-Robin.

The three test methods chosen were: indentation fracture (IF), indentation strength (IS), and single-edge precracked beam (SEPB). These methods are schematically illustrated in Fig. 1. The SEPB and IF methods are standards in Japan and the procedures in this round-robin were in accordance with JIS R 1607 [1].

The IF test is a variant of the scheme originally proposed by Evans and Charles [2]. A polished sample is indented with a Vickers hardness indenter and the length of the resultant median cracks measured. The fracture toughness is related to the indentation load, the size of the median cracks, the elastic modulus and hardness of the material. The test has the virtues that it measures a “micro” fracture toughness, (that is, a toughness relevant to the scale of naturally-occurring defects) and requires only a small amount of material. Drawbacks include the need to rely on a calibration constant to deal with the complex deformation and residual stress fields, and the plethora of equations that have developed for computing fracture toughness by this method as discussed in Refs. [2–6].

The indentation strength (IS) method involves the implantation of an artificial flaw on the surface of a flexure specimen and fracture of the specimen in three- or four-point flexure [7]. A Vickers indentation is used to create the artificial flaw. It is not necessary to measure the initial crack size, since the crack will extend stably during subsequent loading in response to the external load and the residual stress field associated with the indentation. Fracture toughness is calculated from the elastic modulus, indentation load, Vickers hardness and flexural strength.

The single-edge precracked beam (SEPB) method [8,9] is a variation on the traditional single-edge notched beam method. In the latter test, a precrack is formed by a thin saw cut since fatigue precracking is difficult with advanced ceramics. The saw cuts are nevertheless blunt and measured toughness are typically too high. The SEPB method solves the precracking problem by means of a “bridge indentation” scheme, wherein an indented or saw cut flexure specimen is compression loaded in a bridge anvil until a precrack pops in. The precracked beam is then fractured in three-point flexure and the fracture toughness evaluated from an equation by Srawley [10]. The crack size must be measured in some manner, often by dye penetration or by subsequent fractographic analysis. An advantage of this method is that, with the choice of suitable specimen and flexure fixture dimensions, the test is similar to ASTM standard test method E-399, Plane-Strain Fracture Toughness of Metallic Materials [11].

The Japan Fine Ceramic Center accumulated the available results from thirteen laboratories in 1990 and prepared reports summarizing the findings [12–14]. Five U.S. laboratories completed their testing in the round-robin by the summer of 1991 and this report presents their results and findings. The five participating U.S. laboratories are listed in Table 1. These labs will hereafter be referred to as USA labs 1–5.

## 2. Materials

The Japan Fine Ceramic Center furnished all specimens for the round-robin. Two materials were used:

Gas-pressure sintered silicon nitride, Grade EC-141^1^,^2^, (hereafter designated silicon nitride)

Zirconia alumina composite

Grade UTZ-20^1^,^2^, (hereafter designated ZAC).

Twenty specimens of each material were sent to the participants. Specimen dimensions were 3 × 4 × 40 mm. One of the 4 mm wide sides was ground and polished by a #2000 diamond grinding wheel to provide a good reference surface for indentations.

The silicon nitride is a commercial grade sintered silicon nitride that is used for automotive turbochargers [14,15]. Yttria and alumina are used as the sintering aids. The microstructure has fine (1–2 μm), equiaxed β-silicon nitride grains and a glassy boundary phase [16]. The room-temperature flexure strength is approximately 900 MPa. Strength gradually drops to about 60% of this value at 1200 °C [17,18]. Young’s modulus is 310 GPa at room temperature. The gas-pressure sintering process is expected to produce a homogeneous and isotropic material.

**Fig. 2:**
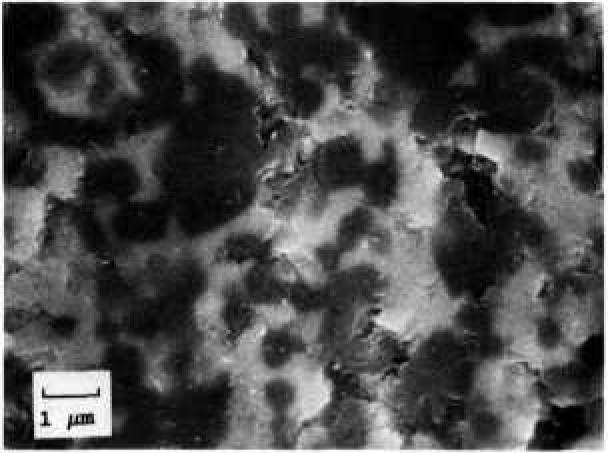
A scanning electron micrograph of the zirconia alumina composite (ZAC). The white phase is zirconia, the dark phase, alumina. Some residual porosity is also evident.

ZAC, a composite with about 50% zirconia and alumina, was fabricated by pressureless sintering. The Young’s modulus was given as 280 GPa. Figure 2 shows the microstructure. X-ray diffraction on the polished surface of a specimen indicated the primary phases are alpha alumina and tetragonal zirconia. Some monoclinic and cubic zirconia were also detected. Energy dispersive spectroscopy on the scanning electron microscope revealed only aluminum and zirconium. Silicon was not detected.

Two important issues regarding advanced ceramic crack growth are whether there are environmental effects and whether the material has *R*-curve behavior. Both these phenomena interfere with the goal of measuring fracture toughness.

**Fig. 3:**
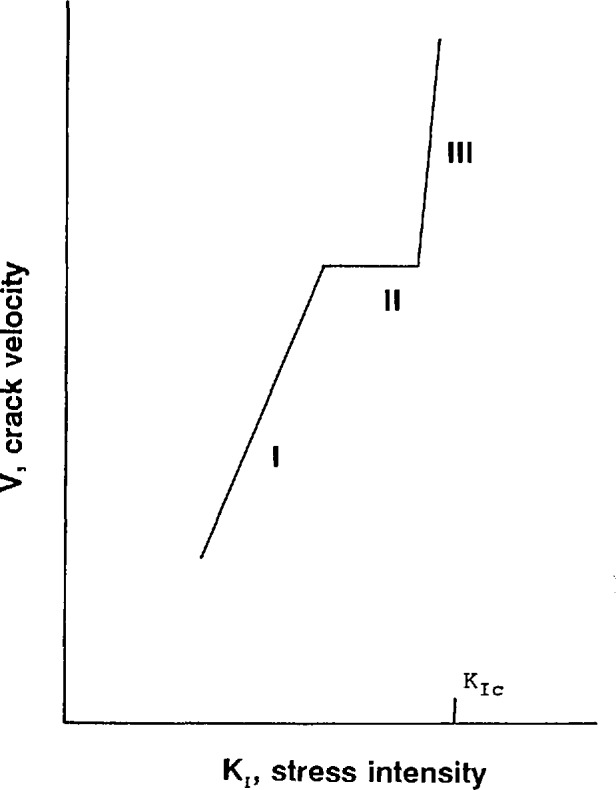
Environmentally-assisted crack growth occurs at stress intensities less than *Ku* and can interfere with measurements of fracture toughness.

Environmentally-assisted crack extension is usually depicted on a *V-K*_I_ graph as depicted in Fig. 3. The conventional interpretation is that Region I and II behavior is controlled by the environment, whereas Region III crack extension is intrinsic to the material [19]. The point is that environmentally-assisted, slow crack growth can occur at stress intensities less than *K*_Ic_ and thereby interfere with attempts to measure the latter. Tetragonal zirconia and zirconia alumina composites are known to have glassy boundary phases and are susceptible to slow crack growth phenomena [20,21].

**Fig. 4:**
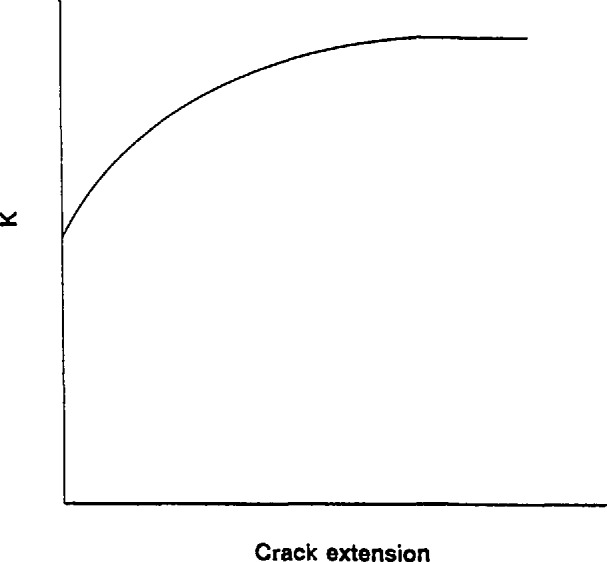
R-curve phenomena can also complicate fracture toughness testing. The resistance to crack extension increases as the crack extends. It is not clear what constitutes *K*_Ic_ in such a material.

R-curve phenomena, illustrated in Fig. 4, are common in advanced ceramics [22–28] and are attributed to interactions of a crack with the microstructure. Resistance to crack extension increases as the crack extends. In advanced ceramics this is often due to wake (behind the crack tip) phenomena such as grain bridging, fiber reinforcement, or dilation from phase transformations. The ZAC with a transformable tetragonal zirconia phase likely will cause rising R-curve behavior, but the fine grain, equiaxed silicon nitride is not likely to do such.

**Fig. 5:**
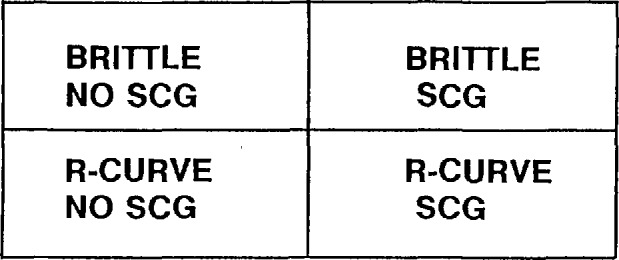
The crack growth behavior of advanced ceramics can be categorized by whether R-curve phenomena, and/or slow crack growth phenomena are active. After Fuller [29].

Figure 5 illustrates one possible framework suggested by Fuller for categorizing advanced ceramics [29]. The simplest condition is a material that has no R-curve behavior (i.e., brittle) and which has no environmentally-assisted crack growth. It is not unreasonable to characterize such a material as having a specific fracture toughness, *K*_Ic_, and test methods could be tailored to measuring such value. It is expected that the silicon nitride will approximate these conditions when tested at room temperature.

If, on the other hand, environmental effects influence crack growth, then results will be very sensitive to the testing conditions, especially the rate of loading and humidity. If R-curve phenomena are active, then a serious question ensues as to what fracture resistance is being measured by a given test. In general, different tests will give different results for toughness, depending upon the precracking history, the amount of crack extension during the test, the amount of crack opening displacement, and the precracking and final loading rates. A material which manifests both environmentally-assisted crack growth and R-curve phenomena poses a formidable challenge, both in testing and interpretation of results [22,23]. The zirconia alumina composite may very well fall into this category.

The test matrix and testing conditions in this round-robin were specified primarily with two objectives: detecting environmental effects through the use of variable loading rates, and observing the sensitivity of the results in the IF and IS methods to the use of different indentation loads. No procedures were specified to detect or quantify R-curve phenomena, although as will be shown, some inferences can be made from the results.

## 3. Experimental Procedure

The experimental procedures were specified in the instructions JFCC furnished to all participants and are summarized below.

### 3.1 Indentation Strength (IS) Method

The polished 4 mm wide surface of each specimen was indented by a Vickers indenter at the individual laboratories. Two loads each were used for the two materials: 49 and 249 N for silicon nitride, and 98 and 490 N for ZAC. Use of two loads permitted an assessment of whether there was a dependence of fracture toughness on indentation load. Ten specimens of each material were indented at each load in the middle of the specimen. The 490 N load for the ZAC is higher than what most microhardness machines can produce, so most laboratories mounted a Vickers indenter onto a universal testing machine and loaded the specimen in a simulated indentation cycle. It is not clear how proper this procedure is and how successfully it was done in the different laboratories. Hardness measurements themselves are notoriously sensitive to loading rate, vibration, and impact. For example, it was very difficult with displacement control machines to simulate the constant load portion of a hardness cycle. NIST held the crosshead stationary for 15 s to simulate that portion of the cycle. NIST was able to control the peak loads to within 1% for 7 of 10 specimens, and within 2.5% for the remaining three. In addition, it was very difficult to control the exact peak load with such machines when they were loading at rates simulating a microhardness machine cycle. During the customary hold time of about 15 s, the crosshead was held stationary and a relaxation of 2% in load was noted for all specimens. In contrast, USA lab 5 unloaded immediately upon reaching the peak load.

The indented specimens were then loaded into a three-point flexure fixture with a 30 mm span, taking care that the indent was loaded in tension directly under the middle load pin. The flexure strength was measured with a crosshead speed of 0.5 mm/min. The specimen and fixture sizes, and the rate-of-loading are consistent with the Japanese flexure strength standard test method: JIS R 1601 [30].

The JFCC instructions specified that the fracture toughness should be calculated by the following equation:

$$K_{\text{Ic}}=0.59\;{\left(E/H_{v}\right)}^{1/8}\;{\left(\sigma_{c}\;P^{1/3}\right)}^{3/4}$$ (1)

where *E* is the elastic modulus, *H_v_* is the *Vickers hardness, σ*_c_ is the flexure strength, and *P* is the indentation load. Unfortunately, the hardness in Eq. (1) is not the same hardness as specified in the original Ref. [7]. Vickers hardness is defined as the load divided by the *actual contact area* of the indenter into the specimen (the surface of the four facets of the pyramidal impression which penetrate into the sample):

$$H_{v}=1.854\;P/{\left(2a\right)}^{2}$$ (2)

where 2*a* is the indentation diagonal size. In Ref. [7], the hardness was defined as the load divided by the *projected area* on the surface:

$$H=2\;P/{\left(2a\right)}^{2}.$$ (3)

The fracture toughness as originally derived in Ref. [7] is:

$$K_{\text{Ic}}=0.59\;{\left(E/H\right)}^{1/8}\;{\left(\sigma_{c}\;P^{1/3}\right)}^{3/4}.$$ (4)

The use of the wrong hardness leads to a systematic error of 1% (calculated values are too high) in fracture toughness if Eq. (1) is used. Equations (3) and (4) are *the proper equations to use for the IS method* as specified in Ref. [7].

### 3.2 Single-Edge Precracked Beam (SEPB) Method

The fractured halves of the IS tested specimens were subsequently used for SEPB testing. Indentations were implanted on the 3 mm wide face, the specimen precracked with a bridge-anvil, the precrack dye-penetrated, the specimen fractured in three-point loading, and the precrack size measured on the fracture surface.

**Fig. 6:**
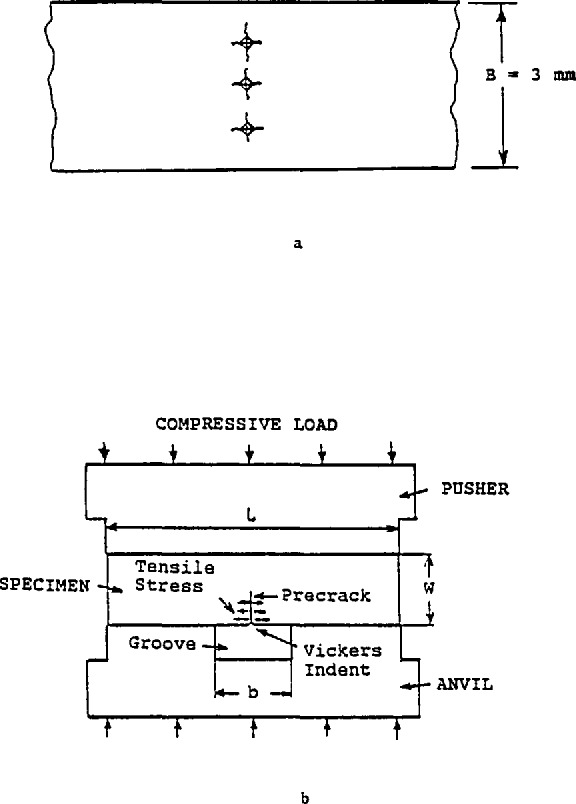
The precracking procedure for the SEPB specimens. Three 196 N indents were used on the ZAC as shown in (a). After indentation, the precrack was popped-in by loading the specimen in a bridge-anvil as shown in (b).

Either an indentation or saw cut can be used as a precursor to the precrack in the middle of the specimen. The JFCC instructions for this round-robin specified use of one Vickers 98 N indent for the silicon nitride, and three 196 N indents for the ZAC as shown in Fig. 6a.

Next, the specimen was inserted into the bridge-anvil as shown in Fig. 6b with the indents located directly over the groove whose dimensions could be varied from 3 to 6 mm as needed. The assembly was inserted into a universal testing machine and load was increased until a precrack popped in. This could be monitored by acoustic emission equipment or by ear. No loading rate was specified, but slow rates are advisable to permit detection of the pop-in and to minimize the risk of load cell over-load. Although not specified in the instructions, once the pop-in load had been established, it was acceptable to preload at a moderate rate up to some fraction of the expected pop-in load (e.g., 80%), and then load slowly until pop-in. Lx)ads between 10000 and 20000 N (2200–4400 lb) were needed for this step, which precluded the use of most small table-top universal testing machines. (NIST utilized a heavy duty machine^3^ for precracking, and a small table-top model^4^ for three-point fracture.) The pop-in load and the precrack length could be adjusted by the selection of different groove widths; the larger the width, the lower the pop-in load and the longer the precrack.

A specific design for a bridge-anvil was furnished by JFCC along with instructions on how to order a set from Japan. Several U.S. participants attempted to acquire such an anvil, but encountered administrative difficulties and ultimately fashioned their own apparatus. This is important since some foreign and U.S. participants had problems obtaining proper precracks with their own designs, whereas the labs using the JFCC design had few such problems. The JFCC reports suggested that improper alignment with the former may have been a problem [12,13].

The depth of the precrack was then measured. The instructions recommended the use of dye penetrants, possibly diluted by acetone. If dye penetrants were used, the instructions specified that the specimens were to be dried at 50 °C for 1 h.

The specimens were then loaded in a three-point flexure fixture with a rather short 16 mm span and loaded to fracture. The specimens were tested at two different crosshead rates (1.0 and 0.005 mm/min) thereby permitting an assessment of whether environmental phenomena affected the results.

**Fig. 7:**
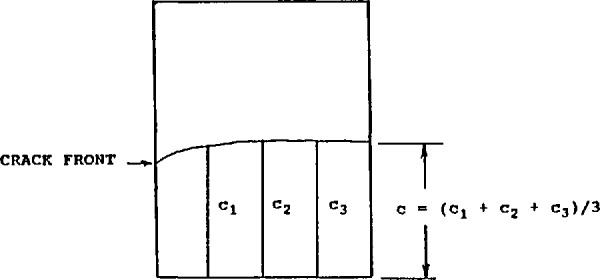
After fracture, the SEPB precrack size was measured on the fracture surface.

After fracture, the precrack length was measured at three locations as shown in Fig. 7. The average of the three measurements was used as the crack length to calculate fracture toughness. The difference between any two of the three length measurements could not exceed 10% of the average, and the plane of the crack had to be perpendicular to the specimen long axis within 10° or else the specimen was rejected. These criteria are from the ASTM fracture toughness standard E 399 [11]. In addition, although it was not clearly stated in the instructions, the precrack length had to be between 1.2 and 2.4 mm. This is a requirement of JIS R 1607 [1], but is not in ASTM E 399 [11].

Fracture toughness was then calculated from Srawley’s equation [13] (which is also the same as in ASTM E 399) which is accurate within ±0.5% for all crack lengths from 0 to *W* [10,11]:

$$K_{\text{Ic}}=\frac{3SP}{2BW^{2}}c^{0.5}\;F\left(\alpha \right)$$ (5)

α=c/W (6)

$$F\left(\alpha \right)=\frac{1.99-\alpha \left(1-\alpha \right)\left(2.15-3.93\alpha +2.7\alpha^{2}\right)}{\left(1+2\alpha \right){\left(1-\alpha \right)}^{1.5}}$$ (7)

where:

*S* is the moment arm of the three-point fixture (16 mm)
*c* is the precrack length
*α* is the normalized precrack length
*W* is the specimen height
*B* is the specimen width
*P* is the load at fracture.

### 3.3 Indentation Fracture (IF) Method

One of the fractured halves of an IS specimen was used for this method. Ten Vickers indentations were placed on the polished surface. Two loads each were used for the two materials: 98 and 196 N for the silicon nitride, and 294 and 490 N for the ZAC. No instructions on loading rate were given. The 294 N load (30 kg) is at the limit of many commercial microhardness testers. As discussed above in the IS section, the 490 N indentation had to be simulated on a universal testing machine and this could have introduced a serious additional source of error or scatter in the IF method. (Microhardness measurements are notoriously sensitive to vibrations and rate of loading.)

The indentation diagonal length, 2*a*, and the crack length, 2*c*, were measured for each impression. If the ratio of the crack length to indentation length, *c*/*a*, was less than 2.3, or if there was crack branching, the data was to be rejected.

Two different equations were to be used for calculation of fracture toughness. Unfortunately, the instructions for the round-robin furnished two forms for each of the equations (for a total of four equations) which led to some confusion.

The derivation by Miyoshi et al. [31] gives:

$$K_{c}=0.018\;{\left(E/H_{v}\right)}^{0.5}\;\left(P/c^{1.5}\right)$$ (8)

$$=0.0264\;E^{0.5}\;P^{0.5}\;c^{-1.5}a.$$ (9)

Equation (9) properly follows from Eq. (8) *if the hardness is H_v_*=1.854*P*/(*2a*)^2^. The instructions for the round-robin specified that Eq. (9) was to be used but also included Eq. (8), but without the subscript “v”. Regretably, some U.S. participants utilized Eq. (8), but with *H* = 2*P*/(2*a*^2^). This leads to a 3.9% error in the IF results. In the results that follow, all data have been corrected to be in accordance with Eq. (9) as specified by the round-robin instructions and by Miyoshi et al. [31].

An alternate equation derived by Marshall and Evans [32] was also prescribed by the round-robin instructions:

$$K_{c}=0.036\;E^{0.4}\;P^{0.6}\;a^{-0.7}\;{\left(c/a\right)}^{-1.5}$$ (10)

$$=0.036\;E^{0.4}\;P^{0.6}\;a^{0.8}\;c^{-1.5}.$$ (11)

In this instance, there is no confusion with hardness since it does not appear. Equations (9) and (11) are very similar and have the same *c* dependence. The *E, P*, and *a* dependencies are slightly different and reflect a small difference in the dependence of the *E/H* ratio used in the original derivations. Dividing Eq. (9) by (11) gives:

$$K_{c},\text{Eq}.\;(9)/K_{c},\text{Eq}.\;(11)=0.689\;{\left(E/H\right)}^{0.1}.$$ (12)

Equations (9) and (11) give values of *K*_c_ that are about 7% different since *E* is constant and *H* varies only a slight amount over the range of indentation loads used. The Miyoshi et al. [31] Eq. (9) gives the smaller value of *K*_c_. (The difference is also about 7% if *H*_v_ is used.)

Several laboratories also measured the Vickers indentations and cracks from the precracking of the IS specimens (described above) and thus were able to obtain additional data for IF analysis. The indentation loads available for IF analysis are shown in Table 2.

## 4. Results and Discussion

The U.S. laboratory results are presented here in the same format as used by JFCC for the earlier partial results. This is done to permit easy comparison of the U.S. results to those of the other participants. The results from the U.S. labs are identified in the figures as U.S. labs 1–5.

### 4.1 Indentation Strength (IS) Results and Discussion

**Fig. 8:**
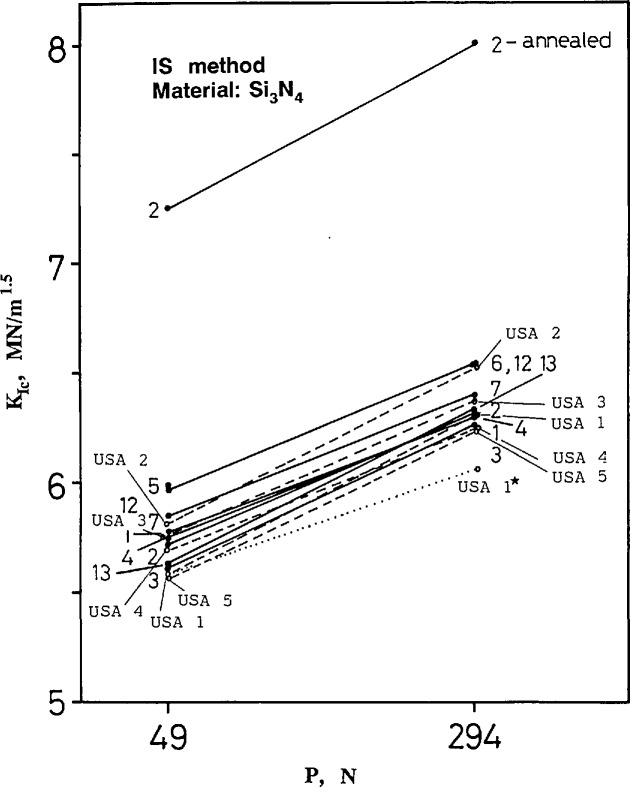
Fracture toughness for the silicon nitride as measured by the indentation strength (IS) method. The dotted line shows the USA lab 1 data for the 294 N indentation load corrected for the stress gradient (USA 1*).

**Fig. 9:**
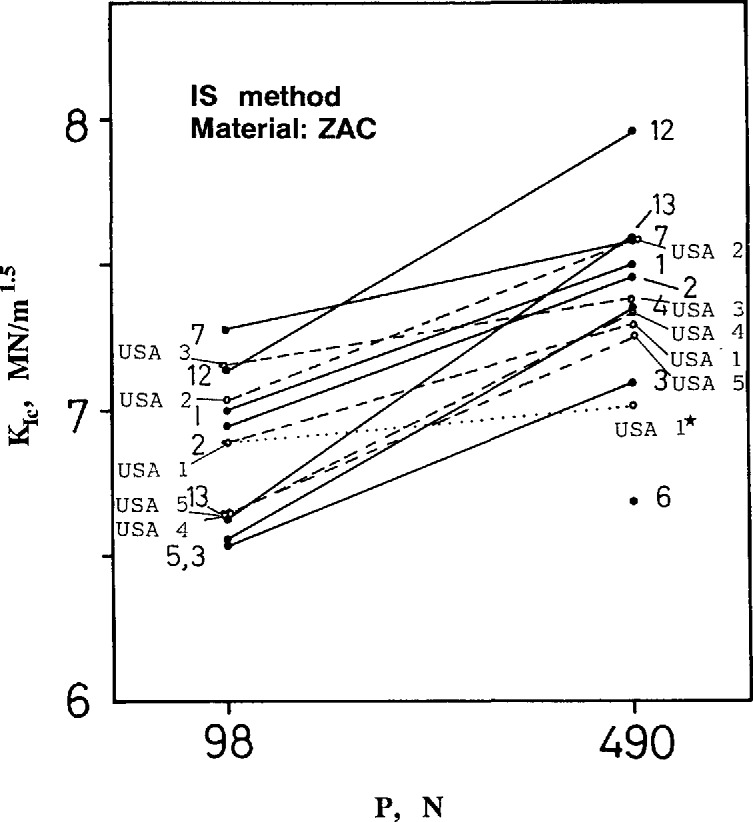
Fracture toughness for the ZAC as measured by the indentation strength (IS) method. The dotted line shows the USA lab 1 data corrected for the stress gradient.

**Fig. A1:**
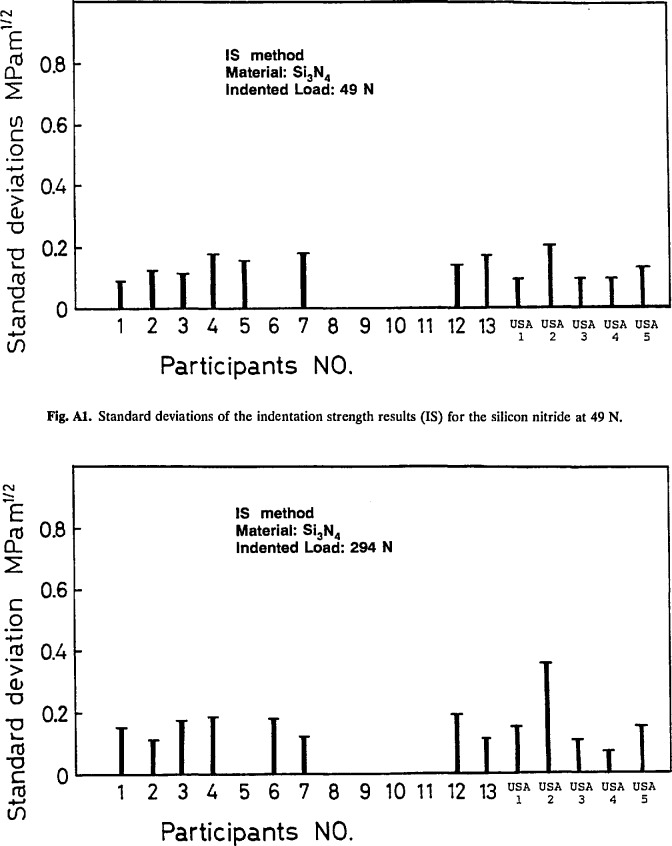
Standard deviations of the indentation strength results (IS) for the silicon nitride at 49 N.

**Fig. A2:**
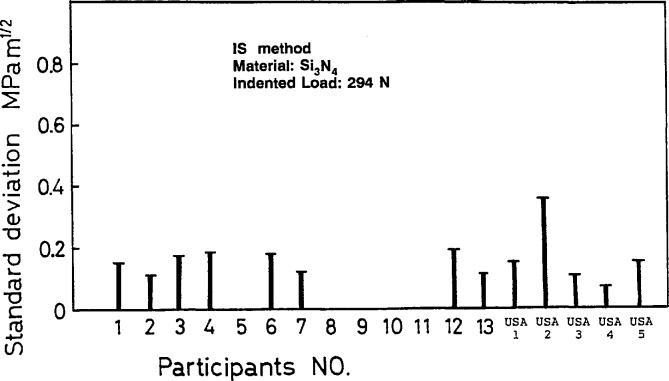
Standard deviations of the IS results for the silicon nitride at 294 N.

**Fig. A3:**
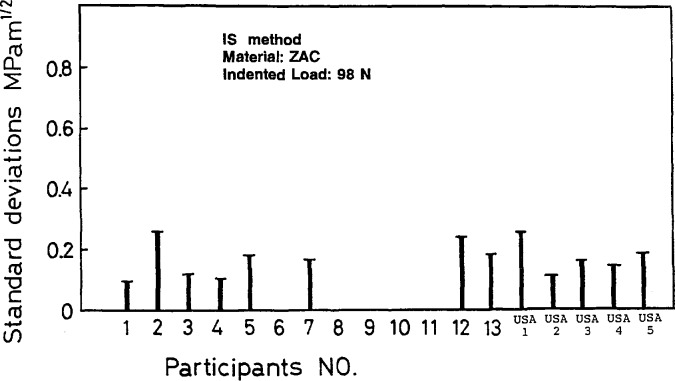
Standard deviations of the IS results for the ZAC at 98 N.

**Fig. A4:**
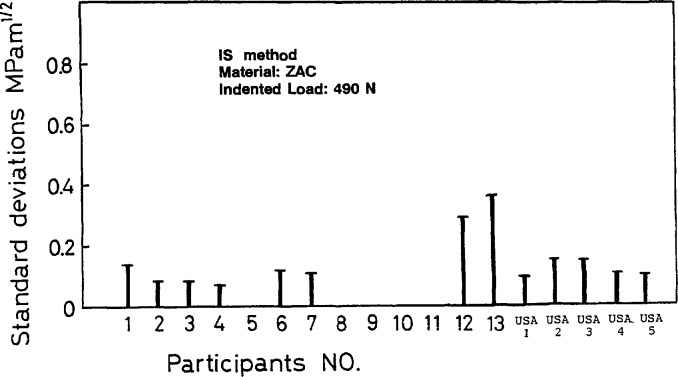
Standard deviations of the IS results for the ZAC at 490 N.

Figures 8 and 9 show the results for silicon nitride and the zirconia alumina composite respectively. The U.S. data are completely consistent with the other data and show the same trend of apparent increasing toughness with indentation load. The within-laboratory standard deviations of the results are also consistent with the other laboratory results and are typically 0.1 to 0.2 MN/m^1.5^ (Figs. A1–A4 in Appendix A). (Foreign lab 2 did not follow instructions and annealed the specimens prior to testing thereby relieving the residual stress. This procedure invalidates the use of Eqs. (1) or (4).) For silicon nitride, the average fracture toughness for all laboratories (excluding lab 2) is approximately 5.8 and 6.3 MN/m^1.5^ at the 49 and 294 N loads, respectively. For the ZAC, the averages are 6.9 and 7.4 MN/m^1.5^, at 98 and 490 N loads, respectively. None of the Japanese labs (8–11) performed IS measurements.

A material with constant fracture toughness would have a fracture load dependent upon the indentation load, *P*, to the minus one third power. Since this dependence is factored into Eqs. (1–4) there should be no dependence of IS toughness on indentation load. The apparent variation in Figs. 8 and 9 could be attributed to a rising “R-curve”, and indeed, techniques to analyze such data have been devised [26–28].

An alternative explanation can be found by noting that the controlled flaw is often quite large relative to the Specimen cross section. The flaw is not exposed to a uniform stress field, but is actually in a gradient which diminishes to zero axial stress at the neutral axis. The stress intensity around a crack can be expressed as:

$$K_{I}=Y\;\sigma \sqrt{c}$$ (13)

where *Y* is the shape factor, *σ* is the far-field stress, and c is flaw depth. The derivation of Eq. (4) assumes that the applied far-field stresses are acting uniformly on the surface crack and that the shape factor, *Y*, for the crack is uniform and remains constant as the crack extends. The shape factor is incorporated into the constant, 0.59, in Eq. (4). An expanded version of Eq. (4) from Ref. [7] is:

$$K_{I}={\left[\left(256/27\right){\left(\pi Y\right)}^{3/2}§\right]}^{1/4}\;{\left(E/H\right)}^{1/8}\;{\left(\sigma P^{1/3}\right)}^{3/4}$$ (14)

where § is a constant for the Vickers produced radial cracks.

Assuming a constant *Y* is only an approximation for surface cracks loaded in bending. Although it is adequate for shallow cracks in large bend specimens, it is more accurate to adjust the stress intensity shape factor, *Y*, for the stress gradient. In recent years, the shape factors corrections derived by Newman and Raju [33,34] have been commonly used. An estimate of the effect of the stress gradient upon the computed fracture toughness is derived below.

The indentation loads used in the present round-robin had to be sufficiently large to ensure fracture from the artificial flaw. Table 3 illustrates that the artificial flaws were quite large relative to the specimen thickness (3 mm). (The surface lengths were measured and the depths shown assume the crack shape is semicircular.)

The initial crack sizes are *not* the crack size at fracture, however. During loading to fracture, the residual stresses from the indentation will combine with the applied external stresses to cause the initial crack, *c*_0_, to extend stably to a size *c*_max_ at which point unstable fracture will occur [7]. The value of *c*_max_ can be estimated from the indentation parameters and, thus, it is not necessary to measure a crack length for this method. (Note that Eqs. (1) and (14) do not include a crack size term.) Not having to measure precrack size is a great experimental advantage. If the plastic indentation zone exerts a constant residual force upon the crack as it extends, the original analysis shows that the precrack will extend 2.52 × its original size in a uniform stress field for a flat R-curve material [7]. The crack extension would be less if the plastic indentation zone behaves as a rigid (fixed-displacement) wedge [27]. The latter study reported experimental extensions along the specimen surface of 2.1 and 2.3× for a SiC-alumina composite and silicon nitride, respectively.

The extent of crack extension into the depth will be much less, however, due to the stress gradient. Thus, the controlled flaw will change shape from a semicircle to a semiellipse. Raju and Newman showed several instances where crack shape changed ellipticity during fatigue growth [33]. Dusza [35] and El Aslabi et al. [36] reported that crack extension was entirely along the surface in ceramic IS specimens, with nearly no extension into the depth. The latter study was on a silicon nitride with similar indentation loads as in the VAMAS round-robin. Ramachandran and Shetty [27], Krause [26], and Anderson and Braun [28] also noted the change of crack shape. Each of these investigators observed that the change in shape would affect their shape factors, but resorted to the use of an average value for *Y.*

**Fig. 10:**
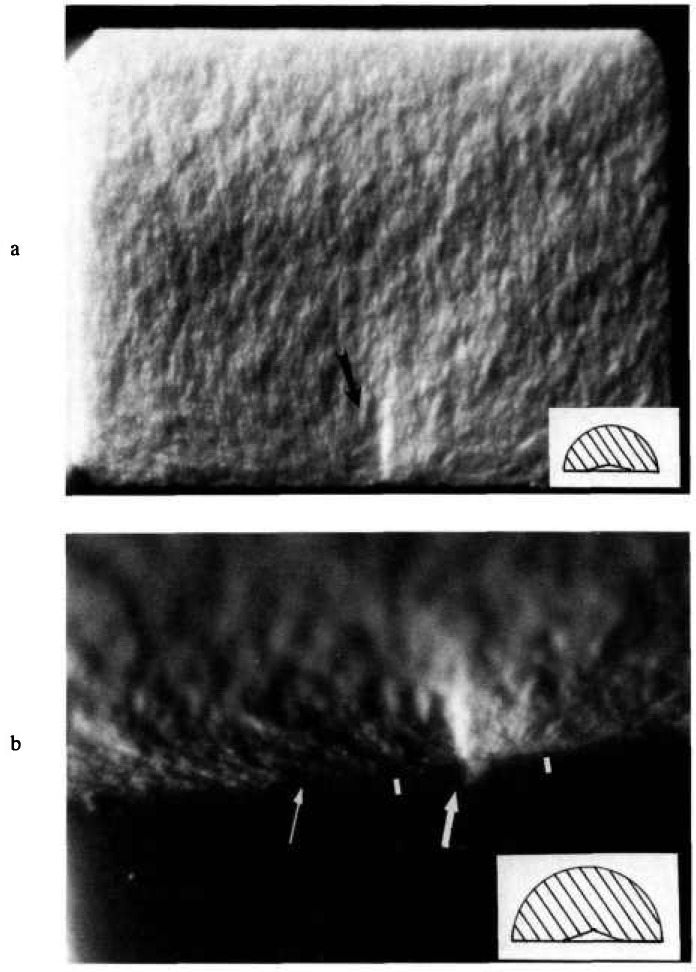
Fracture surface of an indentation strength ZAC specimen with a 490 N indent. An insert in each figure shows (at correct scale) the size of the indentation and the precrack. (a) shows the entire fracture surface. The black arrow points to the area where the indentation fracture origin lies, (b) is a closeup with the specimen tilted to accentuate both the indent and the fracture surface. The large white arrows marks the Vickers indent; the white bars, the precrack; and the small white arrow, the probable extent of stable crack extension prior to catastrophic fracture.

Ideally, the final crack shape at instability could be measured and the correct shape factor could be used in the Eq. (13). The silicon nitride and ZAC specimens tested at NIST were examined to determine if the final crack shape could be measured. The semicircular initial cracks were evident, but the final crack shapes were not clear (Fig. 10). It was therefore necessary to estimate the final crack shape.

The Raju and Newman [33] and most other [34] analyses show that for the semicircular cracks of Table 2, the shape factor, *Y*, is severest at the surface by about 10%. The cracks will grow into semiellipses with an aspect ratio somewhere between 0.7 and 0.9, depending upon the penetration into the flexure stress gradient. Assuming such extension, and taking into account the different initial crack depths of Table 3, it can be estimated from the graphs of Raju and Newman [33] that *Y* for the larger-precracked specimens will be 10% less at fracture than for the smaller-precracked bars. This is for both the ZAC and silicon nitride. The calculated fracture toughness for the larger initial cracks is therefore reduced by *Y*^3/8^ [Eq. (14)], or 4% (relative to the small-load IS specimens). This reduction is shown as a dotted line for the NIST data in Figs. 8 and 9. Much of the apparent dependence of fracture toughness upon indentation load can thus be accounted for.^5^

In summary, the indentation strength (IS) results are quite consistent between the different laboratories, and give an average fracture toughness of 5.7 MN/m^1.5^ for the silicon nitride, and 6.7 MN/m^1.5^ for the ZAC at the lower indentation loads. The scatter in toughness values within each laboratory was quite low, typically 0.1 to 0.2 MN/m^1.5^ The method is simple to conduct and rather popular. One shortcoming of the method is that the true crack shape, the stress intensity factor, the simplifications of the elastic-plastic analyses for the residual stress driving force, and the assumptions of the general similitude of the indentation patterns from material to material, are all embodied in the constant 0.59 in Eq (4). This value was empirically derived by comparison of indentation strength results to results on “standard” materials of “known” toughness. Finally, the specification that three-point loading was to be used added an additional, unnecessary complicating factor in that the precrack had to be precisely located in the three-point flexure fixture. Four-point testing would have been much easier, and possibly more accurate.

### 4.2 Single-Edge Precracked Beam Results and Discussion

**Fig. 11:**
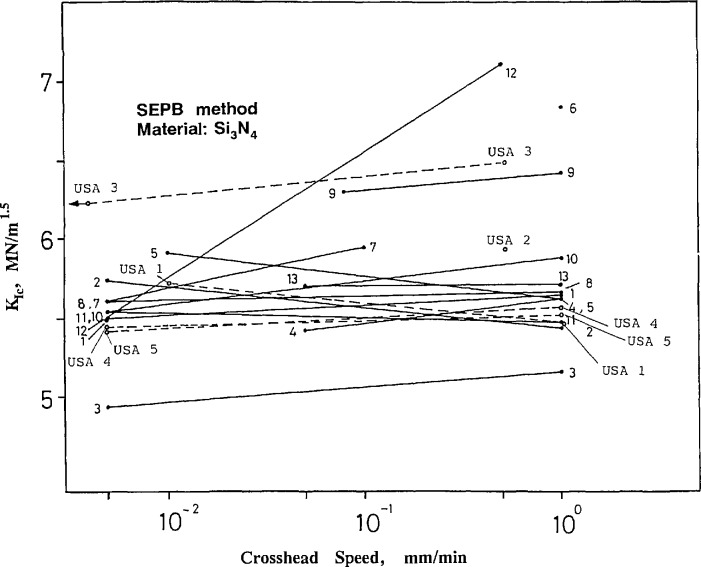
Fracture toughness for the silicon nitride as measured by the single edge precracked beam (SEPB) method. There is a negligible loading rate effect indicating that environmentally assisted crack growth is not a factor.

**Fig. 12:**
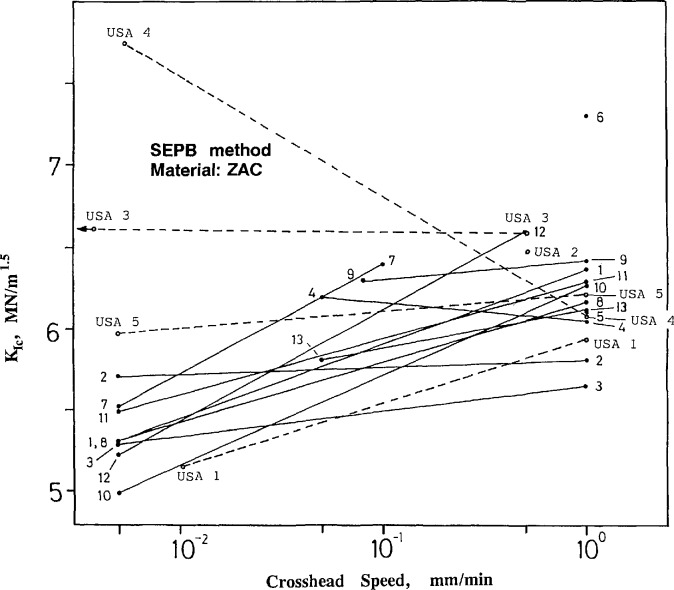
Fracture toughness for the ZAC as measured by the single-edge precracked beam (SEPB) method. The rate dependence of fracture toughness probably is a consequence of R-curve and environmentally assisted crack growth phenomena.

Figures 11 and 12 show the results obtained from the SEPB method as a function of crosshead rate. The use of two widely different crosshead rates permits an assessment of whether environmentally-assisted crack growth was a factor. Most of the laboratories used the recommended speeds of 1 and 0.005 mm/min. USA lab 2 overlooked this, however, and tested all 20 specimens at a single rate. The silicon nitride is, for the most part, insensitive to the rate of loading, whereas the ZAC exhibited pronounced sensitivity. If the fracture toughness is lower at the lower loading rate, the usual interpretation is that environmentally assisted slow crack growth is active. This interpretation will be reconsidered below.

**Fig. A5:**
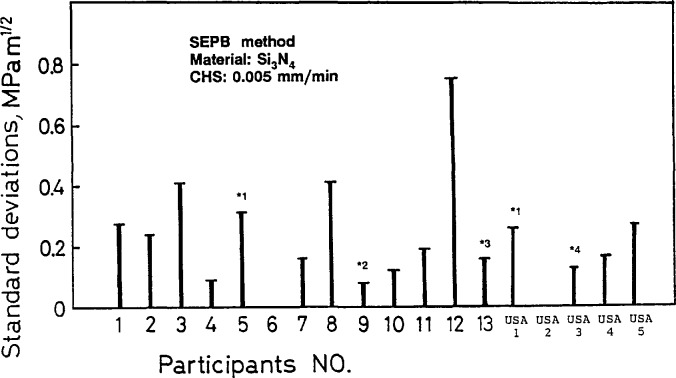
Standard deviations for the SEPB results for the silicon nitride at the slow loading rates. *1. Crosshead speed = 0.01 mm/min. *2. Crosshead speed=0.08 mm/min. *3. Crosshead speed = 0.05 mm/min and eight specimens only. *4. Crosshead speed=0.0025 mm/rain.

**Fig. A6:**
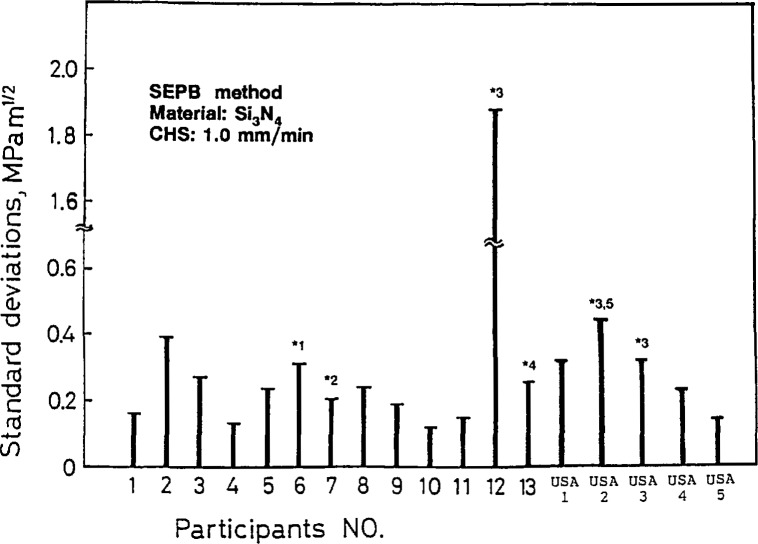
Standard deviations for the SEPB results for the silicon nitride at the fast loading rates. *1. Span = 15 mm. *2. Crosshead speed = 0.1 mm/min. *3. Crosshead speed = 0.5 mm/min. *4. Seven specimens only. *5. 18 specimens.

**Fig. A7:**
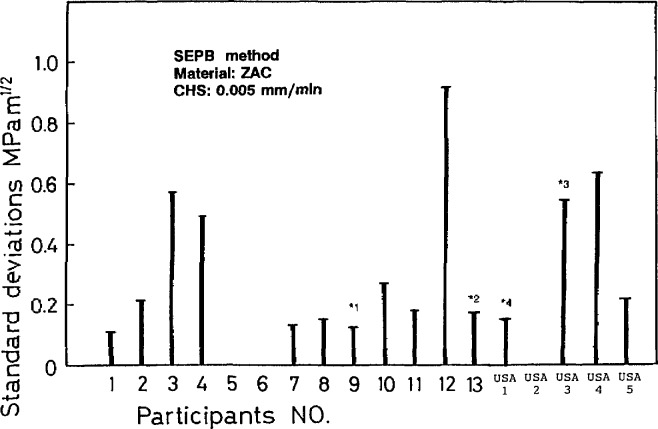
Standard deviations for the SEP5 results for the ZAC at slow loading rates. *1. Crosshead speed = 0.08 mm/min. *2. Crosshead speed = 0.05 mm/min and seven specimens only. *3. Crosshead speed = 0.0025 mm/min. *4. Crosshead speed=0.001 ram/min.

**Fig. A8:**
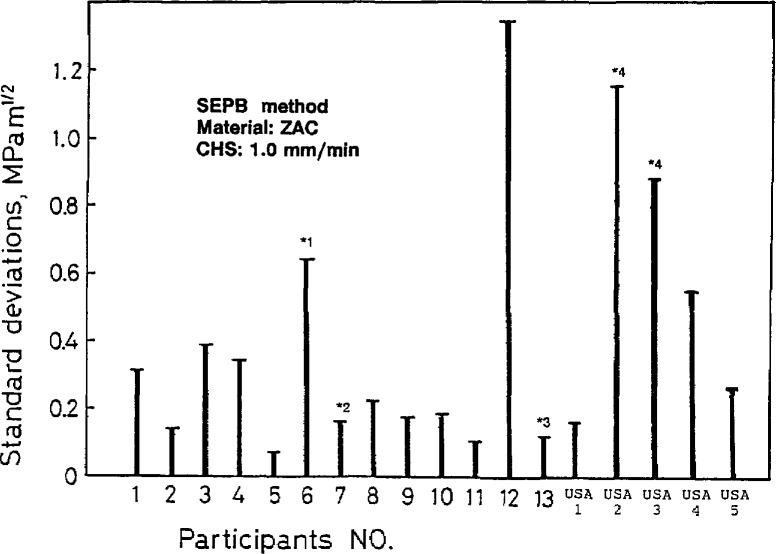
Standard delations for the SEPB results for the ZAC at fast loading rates. *1. Span = 15 mm. *2. Crosshead speed = 0.1 mm/min. *3. Seven specimens only. *4. Crosshead speed 0.5 mm/min.

The toughness values for the silicon nitride are, for the most part, in very good agreement between the laboratories and cluster about 5.6 MN/m^1.5^. The standard deviations within the all labs are usually between 0.1 and 0.4 MN/m^1.5^. The U.S. laboratories are between 0.1 and 0.3 MN/m^1.5^ as shown in the figures in the appendixappendix. The exceptions are USA lab 3 and labs 3, 6, 9, and 12 from the original reporting labs. Of the four Japanese labs (8–11), labs 8, 10, and 11 had very consistent results with low scatter (Figs. A5–A8 in Appendix A), but lab 9 seemed to be systematically high.

The ZAC results have higher scatter as shown in Fig. 12. Most results are within a range of 0.75 MN/m^1.5^ and show the trend of higher toughness with higher loading rate, although sometimes, *individual* labs (such as labs 2 and 4, and USA labs 3 and 4) concluded there was *no* such dependence. USA lab 4 had drastically divergent results and it is tempting to conclude the results are erroneous. They are not, and USA lab 4 discerned an important phenomenon that raises important questions about the interpretation of the SEPB test results which are deferred until later in this section.

**Fig. 13:**
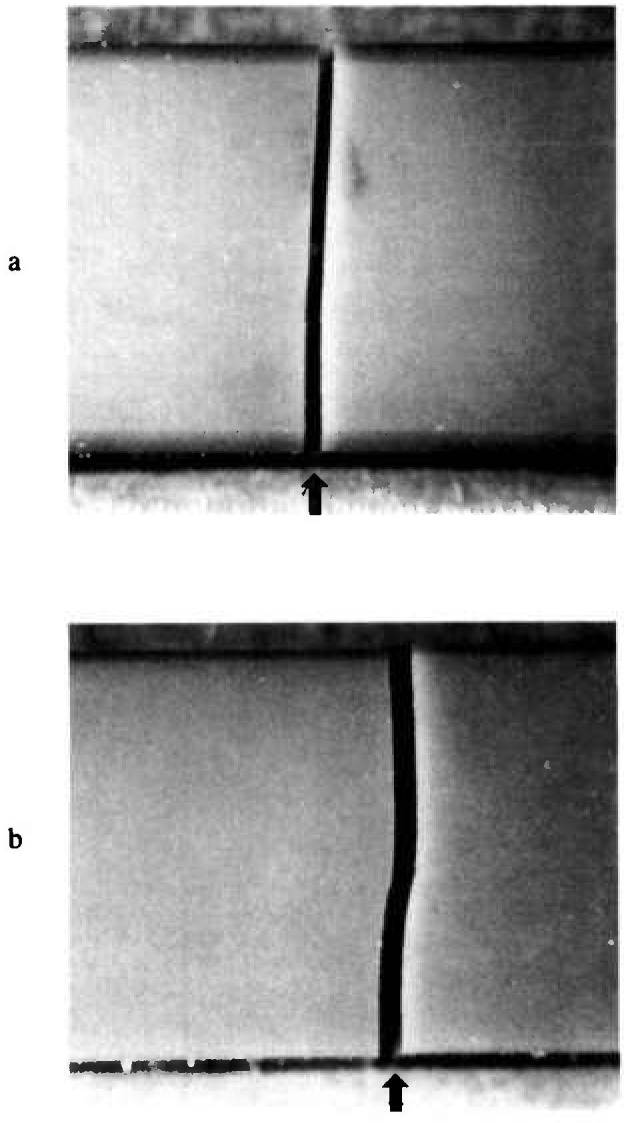
side views of fractured silicon nitride SEPB specimens. The arrows mark the precrack.

The initial reports by JFCC on the round-robin suggested there may have been confusion and problems by some of the participants who had never tried the SEPB test method before [12,13]. One problem proved to be in measuring the precrack size, a task which required some experience and skill. Several labs that were unfamiliar with the bridge-anvil precracking method reported difficulties in obtaining properly precracked specimens. Figure 13 shows two silicon nitride SEPB specimens: one with and one without straight precracks. Alignment of the homemade bridge-anvils is the probable source of the difficulty. JFCC suggested that some of the precracking jigs may not have been adequate to the task. On the other hand, there were sufficient fragments left over from the IS testing that a large number of specimens could be tried until an adequate number of SEPB specimens could be tested. The high compression loads also posed a severe risk of universal testing machine or load cell damage if the operator inadvertently pressed a wrong crosshead speed button.

The flexibility of precracking conditions led to some differences in precise procedures with unknown attendant effects on the final results. For example, the rate of loading was unspecified in the instructions: “Increase the load gradually until a pop-in sound is detected by ear or a sonic sensor.” Some laboratories used older, screw-driven machines and crosshead rates had to be kept low to hear the pop-in against the background machine noise. Other laboratories with quieter machines precracked at faster rates.

NIST and USA lab 3 (at least initially) used a stethoscope attached to the bridge-anvil support to detect pop-in. USA labs 3 and 5 visually observed precracking through a hole in the side of the bridge-anvil at the same time that the crack was exposed to a dye penetrant. Most other laboratories applied dye penetrant *after* precracking. The use of a dye penetrant *during* precracking may alter the precracking process by enhancing intergranular, environmentally assisted crack growth as opposed to stable fast crack pop-in. The rate of precracking has been shown to have a strong effect upon the type of precrack (trans-versus intergranular) and upon the final toughness result in materials with R-curve producing microstructures [8,37].

The silicon nitride specimens precracked with a distinct (albeit faint) pop and it is believed that the material was less sensitive to details of precracking than the ZAC. USA lab 5 used load control (rather than displacement control) and reported that it was easier to detect a distinct pop-in at *higher* loading rates. NIST used a slow loading rate for the ZAC and observed (by interrupting the procedure, removing the specimen, and dye penetrating it) that precracking commenced at loads as low as 9000 N, and stably propagated in short extensions (with attendant sound emissions) as load was increased. NIST and USA lab 3 discerned a series of faint snapping noises during the ZAC precracking. The evidence strongly suggests R-curve phenomena. Differences in precracking may have affected the ZAC results as will be discussed below.

**Fig. 14:**
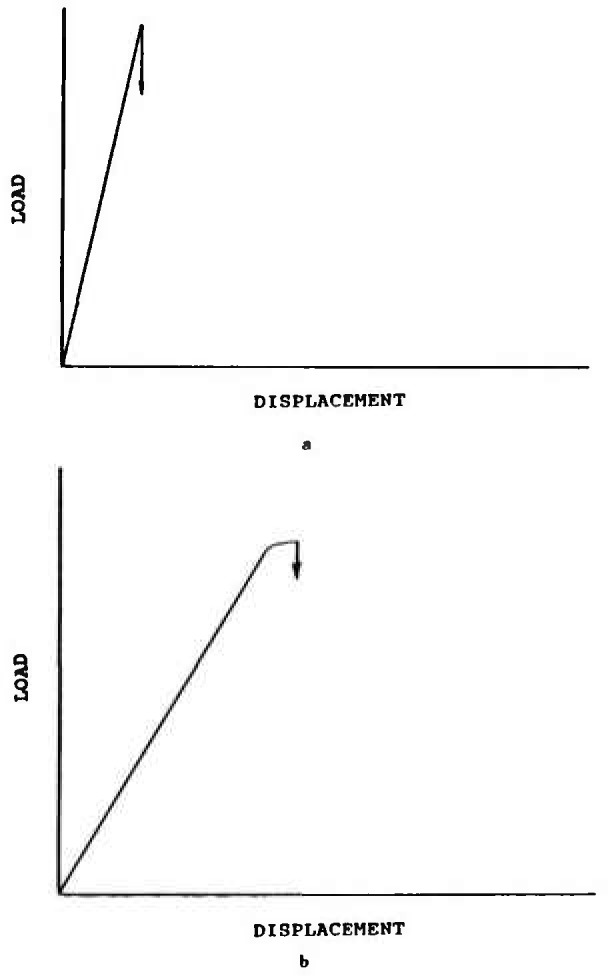
Silicon nitride SEPB specimens at both loading rates, and the ZAC at the fast loading rate, had linear loading to fracture as shown in (a). The ZAC at the slow loading rates had several seconds of stable crack growth prior to catastrophic fracture as shown schematically in (b).

After precracking, the specimens were loaded to fracture in three-point flexure. The silicon nitride at all loading rates, and the ZAC at the fast rates, exhibited an essentially linear load-deflection curve as illustrated in Fig. 14. NIST and USA lab 4 reported that the ZAC, in contrast, had a slight non-linearity at the slower loading rates as illustrated in Fig. 14. This is an important observation and means that the *crack grew stably prior to catastrophic fracture.* The stable growth can be interpreted as coming either from environmentally assisted crack growth, or from rising *R*-curve behavior. The environmental growth probably does not fully explain the observed results since, for a flat R-curve material, any crack extension in a single-edge loaded beam usually leads to unstable-crack extension (for “soft” testing machines and fixtures, and for the precrack sizes of the specified ranges of this round-robin^6^.) Therefore, rising R-curve phenomena probably exists in the ZAC.

One minor observation at NIST during the three-point loading was that alignment of the precracked beam in the three-point fixture was a nuisance. The SEPB specimens had a very short length (~20 mm) and were tested on a 16 mm span. A 1% error in positioning the precrack is only 0.08 mm (0.003 in)! It is not known what effect such misalignments had upon the measured values of toughness. Four-point fixtures would have been easier to use and would have eliminated this potential error source.^7^

**Fig. 15:**
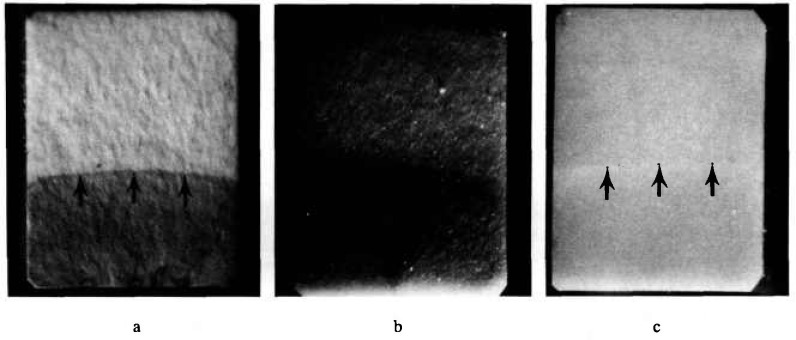
Fracture surfaces of ZAC (a) and silicon nitride (b) SEPB specimens. The colored penetrant on the ZAC is quite definitive. The silicon nitride was more difficult to dye penetrate and low-angle incident lighting was necessary. The position of the shadow in (b) could be altered significantly, however, depending upon the lighting angle. Considerable care had to be taken to make proper size measurements as marked by the arrows in (c).

After fracture, all labs measured the “precrack” size on the fracture surfaces and rejected any specimens where the crack was misaligned, uneven, or not in the specified size range. Measurements were made using optical microscopes or photos taken with a microscope. Figure 15a shows a typical precrack in the ZAC. Table 4 shows some of the procedures used by the U.S. labs. Each lab developed a procedure after a few trial and error steps. The easiest and most reliable method for the ZAC was application of a felt-tip pen to the precrack which stained the white material quite effectively. The opaque silicon nitride was much more problematic (Figs. 15b,c) and none of the dye penetrants worked effectively. Most bled during subsequent fast fracture and storage which led to false crack length measurements. Two labs reported this occurred despite drying cycles or protracted periods of storage prior to fracture. The best procedure for the silicon nitride was optical microscopy with low-incident angle lighting.

The apparently unusual results of USA lab 4 for the ZAC (Fig. 12) can now be reexamined in light of the known R-curve phenomena and of the USA lab 4 precracking procedure. Their procedure for staining the precrack was unsuccessful and they resorted to measuring the “precrack” by low-angle incident lighting. The ridge or feature that they observed in the ZAC is almost certainly *not the original crack from the precracking step, but instead, the crack length at the point of instability.* This crack length was considerably longer (~0.4 mm) than the pop-in crack size because of stable crack extension during the slow loading to fracture. The longer precracks that USA lab 4 used in Eqs. (5–7) account for their apparently high values of fracture toughness.

**Fig. 16:**
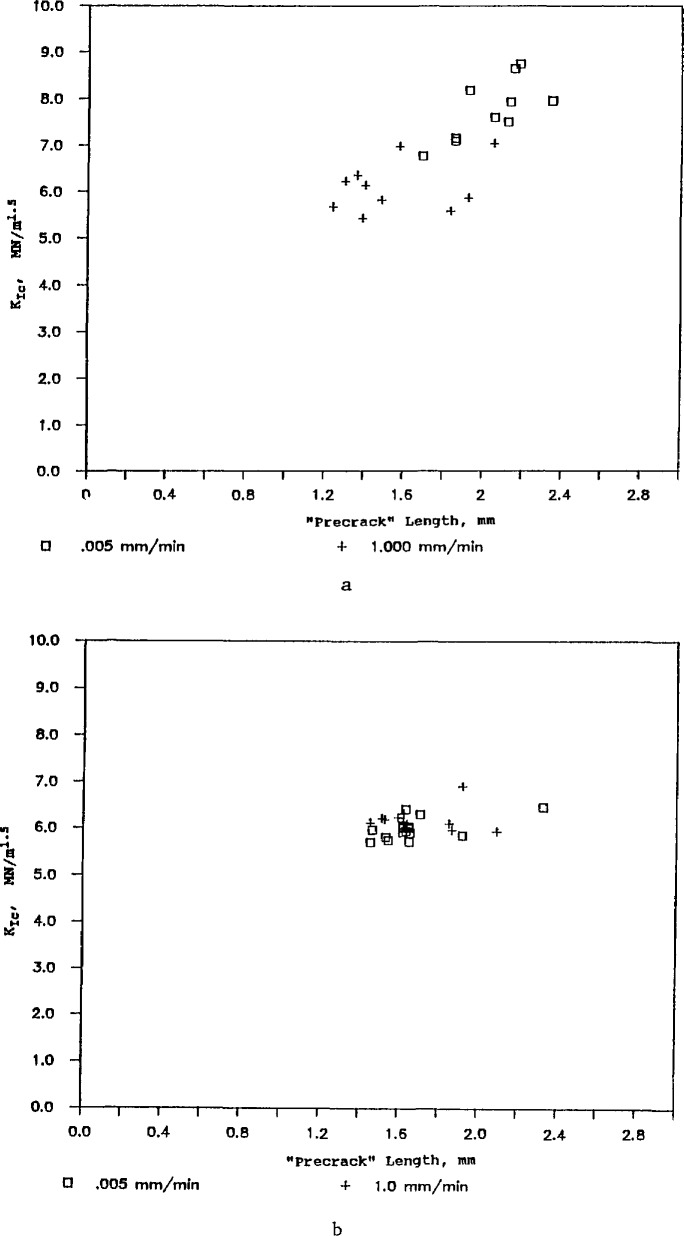
Apparent SEPB fracture toughness versus “precrack” size for the ZAC. (a) is from USA lab 4, and (b) is from USA lab 5. The precrack lengths in (a) are the initial crack lengths after bridge anvil precracking; whereas those in (b) represent the crack length at instability.

This is further reflected in Fig. 16 which shows the apparent fracture toughness as a function of crack size for USA labs 4 and 5. USA lab 4 concluded there was a strong dependence of fracture toughness on crack length and USA lab 5 concluded there was none.

This raises a rather fundamental question about what crack size is appropriate for the computation of fracture toughness for the ZAC: the pop-in precrack length or the crack length at instability? Indeed, this raises the additional question of what point on the R-curve (Fig. 4) does the measured fracture toughness lie.

One aspect of SEPB testing that was not addressed in the round-robin is the possible residual influence of the indents upon the final results. The indents are intended to act strictly as precrack starters and are presumed to have no result on the final fracture. Several investigations have concluded that this is not correct, and that proper results are only obtained if the indentations and their residual stress fields are removed prior to testing [40–43]. Further work is warranted to further clarify whether this is true only for short precrack lengths.

In summary, for the SEPB method, very consistent results were obtained by four of the five USA labs for the silicon nitride. The results were in agreement with the bulk of the other reported data from the international participants. There were negligible effects from loading rate or R-curve phenomena. The ZAC results were somewhat less consistent. Interpretation is severely complicated probably by both R-curve and environmentally assisted crack growth phenomena. The meaning of the measured fracture toughness in this material is unclear.

### 4.3 Indentation Fracture (IF) Results and Discussion

**Fig. 17:**
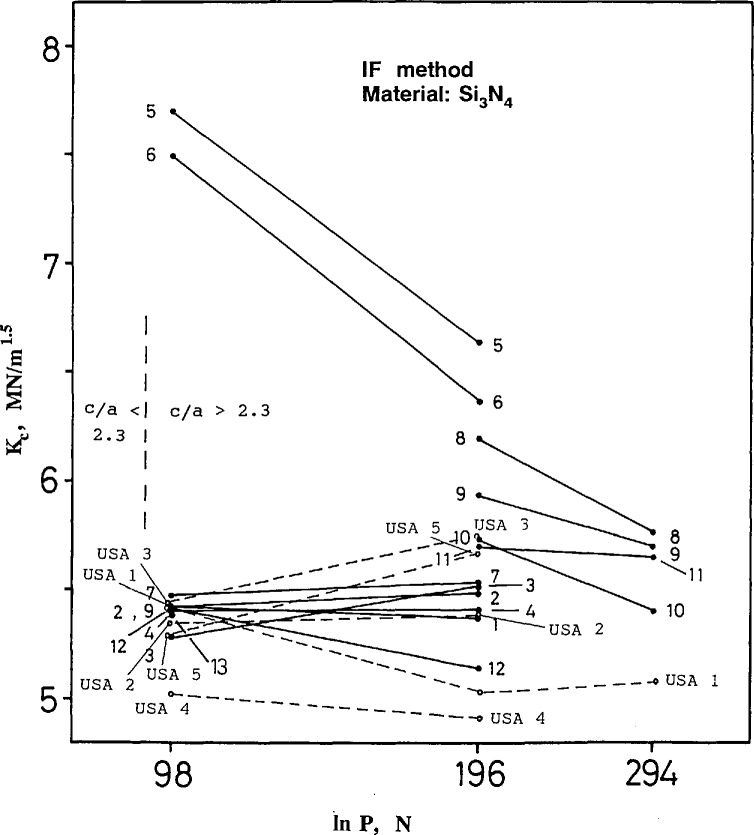
Indentation fracture (IF) fracture toughness for the silicon nitride.

**Fig. 18:**
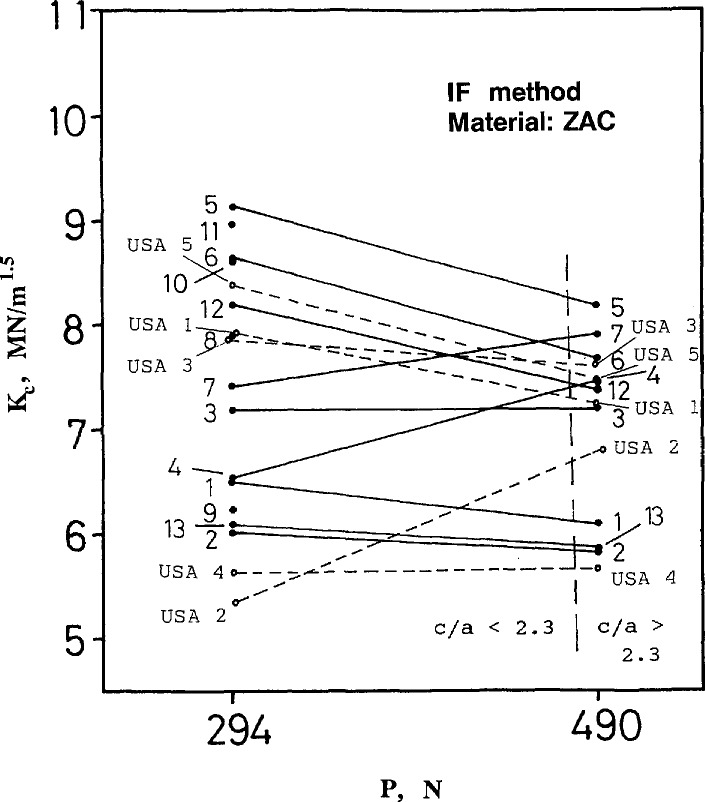
Indentation fracture (IF) fracture toughness for the ZAC.

**Fig. A9:**
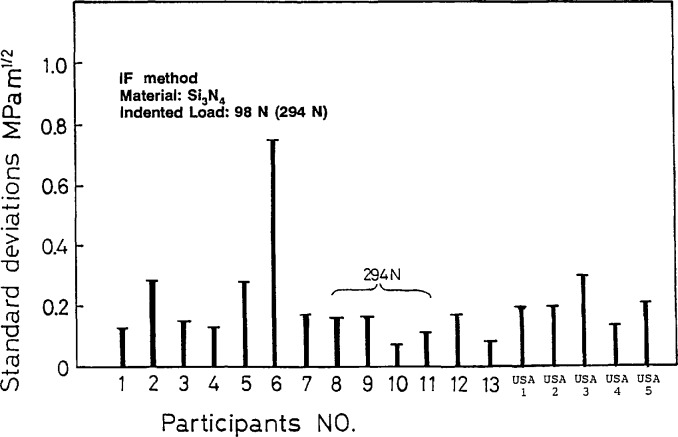
Standard deviations for the indentation fracture (IF) results for tlie silicon nitride at 98 or 294 N loads.

**Fig. A10:**
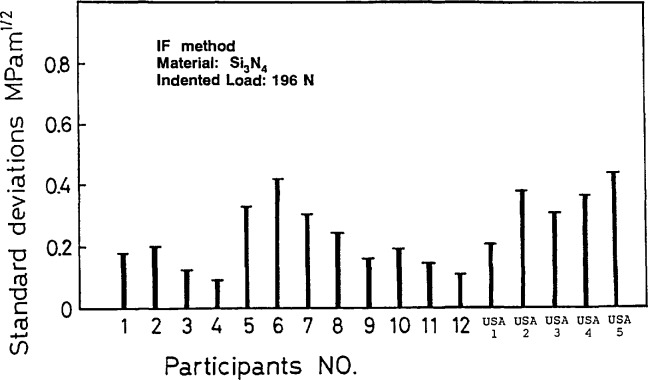
Standard deviations for the IF results for the silicon nitride at 196 N.

**Fig. A11:**
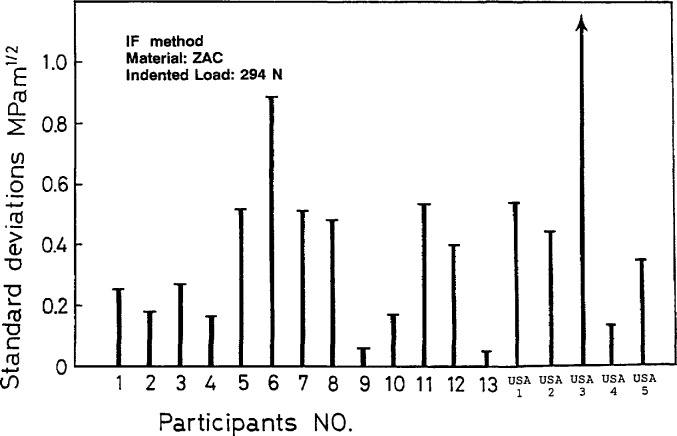
Standard deviations for the IF results for the 2:AC at 294 N.

**Fig. A12:**
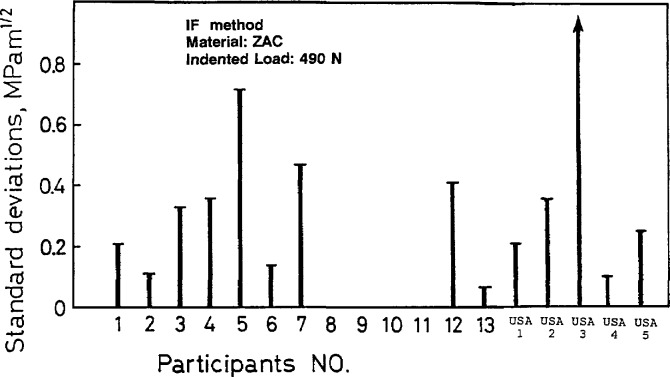
Standard deviations for the IF results for the ZAC at 490 N.

The IF results for silicon nitride and ZAC are shown in Figs. 17 and 18, respectively. The scatter in these results is shown in the appendix as Figs. A9–A12.

The USA lab results for the silicon nitride are consistent with the main body of data. There was no explanation for the very deviant results from foreign labs 5 and 6 [12–13]. The four Japanese labs 8–11 obtained systematically higher toughness values than the other participants. (The scatter in results within an individual lab are typically 0.15–0.3 MN/m^1.5^, so the differences shown in Fig. 17 are systematic.) High values of fracture toughness correspond to shorter measured crack lengths.

The IF results for the ZAC are widely scattered and it is not possible to dismiss the results of any laboratory as being deviant. Toughness ranged from 5.3 to 9.2 MN/m^1.5^. Scatter in results within each lab varied widely from as low as 0.1 MN/m^1.5^ to as high as 1.3 MN/m^1.5^.

Both Eqs. (9) and (11) show that fracture toughness depends upon crack length, *c*, raised to the minus 1.5 power. A ±10% variation in *c* therefore causes a +17 to −13% variation^8^ in *K_c_* or a net scatter (ratio) of 1.17/.87 = 1.34. This variability probably accounts for most of the scatter in the results. The ZAC scatter at 294 N is from 9.2 to 5.3 MN/m^1.5^, =1.7, and the silicon nitride at 196 N is from 6.6 to 5.0 MN/m^1.5^, =1.32.

Several USA labs reported that the crack lengths measured were highly dependent upon the mode of viewing. All labs observed that there was considerable interpretation as to where the exact crack tip was and that there was difficulty in measuring this point. Different viewers were apt to obtain different results on the same specimen. Most agreed that the optics furnished with the microhardness machines were woefully inadequate for measuring crack lengths. (Most used more powerful microscopes.) NIST utilized a reflected light microscope’ at up to 400× with a video camera connected to a 32 cm television monitor. Crack length measurements were made on the monitor with a mouse-driven set of cross hairs and length measuring software. The software included calibration corrections for the video system. This enabled accurate measurements to be taken in a very short time with minimum effort. A few silicon nitride measurements were repeated on a different optical system, also equipped with a video monitoring system, but without the measurement software. Measurements from the two systems agreed within 3–4%. USA lab 4 reported that their experience was that measurements taken by a scanning electron microscope were typically 10 μm longer than optical measurements.

The indentation loads prescribed by the round-robin instructions were intended to produce cracks sufficiently long that the assumptions entailed in the derivations of Eqs. (9) and (11) would be valid. If the ratio of crack length to indentation diagonal size ratio (*cla*) was less than 2.3, the data were to be ignored. Approximate locations for this threshold as determined with the NIST data set are shown as dashed vertical lines in Figs. 17 and 18. It is evident that the indentation load of 98 N was marginal for the silicon nitride, and the load of 294 for the ZAC was unacceptable. The instructions specified that 10 measurements be made, and only those indentations that were satisfactory would be used.

The need for a *c*/*a* > 2.3 comes from the requirement that the cracks be fully developed median (and not Palmqvist) cracks. A number of recent studies (e.g., [44]) have carefully studied subsurface crack morphologies and report that the transition from Palmqvist to a median crack form can actually occur at ratios as high as 3.0.

There were dramatic differences in whether this *c*/*a* criterion was met from lab to lab since they were measuring different crack lengths for a given set of test conditions. Table 5 shows how the USA labs responded. The international participants also had wildly mixed results on meeting this criterion [12,13]. None of the Japanese labs (8–11) reported results at 98 N for the silicon nitride, suggesting that all their *c*/*a* ratios were <2.3.

**Fig. 19:**
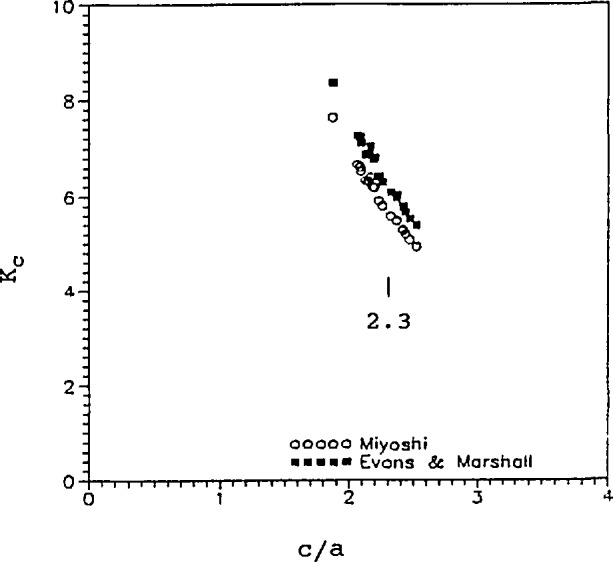
The (IF) fracture toughness varied strongly with the *c/a* ratio for the silicon nitride indented at 98 N. Data and figure from USA lab 5.

Statistical bias problems can arise in situations such as this, depending upon the sampling procedures. The average value can be quite different if (a), of the 10 indentations, only those that meet the criterion are accepted, as opposed to (b), indentations are repeatedly made *until* 10 acceptable patterns are made. (For example, consider representativeness of taking the average of 10 valid indentations if, in fact, 300 indentations had to be made overall.) USA lab 5 reported that there was a pronounced dependence of fracture toughness on the *c*/*a* ratio as shown in Fig. 19. This was the case for both materials and at all indentation loads. Taking only those values for which *c*/*a* > 2.3 leads to using only the lower values of fracture toughness, leading to a clear bias.

There are several complications to the IF method including environmentally assisted crack growth, the decelerating nature of the crack, and R-curve influences. Environmentally assisted crack growth, which can occur in response to the residual stress, tends to make the cracks longer than they would otherwise be. This would lead to underestimates of the fracture toughness. As discussed above in the SEPB results, there seems to be negligible environmental effects for the silicon nitride. Environmentally assisted slow crack growth may be active in the ZAC, but it was difficult to distinguish the rate effects from the R-curve phenomenon. USA lab 5 reported that ten specimens of each material were indented with a drop of oil over the indent but *no difference* in crack lengths was noted.

The conventional interpretation is that the IF median cracks form during the unloading sequence [7], but some instances of formation during the loading have also been reported [45]. In either instance, the crack opens up from, and extends away from the indentation impression until it arrests. Polycrystalline materials have the potential for the microstructure to interfere with the decelerating IF crack, whereas in most fracture mechanics test methods the cracks are accelerating at critical load.

This difference may tend to make the IF cracks shorter than they otherwise would be (if the microstructure were amorphous or very fine and homogeneous) and overestimates of fracture toughness may result from IF testing.

In conclusion, the IF results were disappointing primarily because of the high scatter and failure to obtain consistent interlaboratory results. The strong dependence of the computed fracture toughness upon the crack length (*c*^−1.5^), and the difficulty in measuring such, combined to cause high scatter. Refinements to the measurement technique in principle could improve the accuracy of this method.

## 5. Summary

Table 6 summarizes the apparent fracture toughness values for the different methods. These numbers are estimates based upon the “average” values from the figures presented previously with emphasis on the most reasonable test conditions. The concurrence of values at about 5.5 MN/m^1.5^ for the silicon nitride is encouraging. It is plausible that this material has a constant fracture toughness (flat R-curve) and a negligible loading rate dependence. The variability in the estimates for the ZAC probably reflects R-curve and environmentally assisted crack growth phenomena. The different test methods may be giving fracture toughness values corresponding to different points on the R-curve.

The scatter in IF results for the silicon nitride shows that any one lab could stray typically as much as 0.5 MN/m^1.5^ off the mainstream results, although in some instances there was even more deviation. The scatter in IF results for the ZAC, both within a lab and between labs, was so high as to render the results highly suspect. The effects of R-curve phenomena upon the IF test method values are uncertain.

The IS method had lower scatter and very plausible results for the silicon nitride, making this a very attractive, simple laboratory test for estimating fracture toughness. The ZAC results also had a relatively low scatter, about equal to that obtained by SEPB. Once again, the R-curve and environmentally assisted crack growth phenomena had an uncertain effect upon the ZAC results. Either low indentation loads (for small crack sizes) or large specimen cross-sections are recommended for IS testing to minimize the stress gradient problem. Four-point loading may be preferable for small specimens.

The generally consistent results obtained on the SEPB method for the silicon nitride are also encouraging. Some questions, both in testing procedure and in interpretation, were again raised about using this method for the ZAC in the context of R-curve behavior. Careful attention needs to be placed on specifying exactly which “precrack” length should be measured in such instances. Testing in four-point loading would simplify the SEPB procedure.

## 6. Conclusions

Table 7 summarizes the fracture toughness test methods that are normally used by the participating USA labs. The indentation fracture (IF) method is not commonly used and several laboratories complained that interpretation of the method is “ambiguous.” All agreed that it is difficult to accurately and precisely measure the cracks, that there is significant variability between observers. Four of the five labs felt the method was not reliable. The method is not suitable for elevated-temperature testing. The high scatter in the results of the present round-robin indicate that, at the least, better procedures for measuring the cracks are necessary. The participants for the most part felt that the method may be adequate in the laboratory as a research tool, but is not suitable as a standard for general engineering purposes. These findings are consistent with those of Binner and Stevens in their review paper on this method [46].

The same distrust about the indentation cracks seems to be held by three of the five labs towards the indentation strength (IS) method. This method is widely cited in the ceramics literature, and is felt to provide a good estimate of fracture toughness despite a concern with its empirical roots and “calibration” constants. The method is not applicable to high-temperature testing. The experimental ease of the method (indent and break, without the need to measure cracks) and the fairly consistent results obtained in this round-robin may encourage the broader use of this method as a simple, fast means of estimating fracture toughness for quality control or comparison purposes.

There was a generally favorable reaction to the SEPB method. Three of the labs routinely use it despite its recent development. One other lab reported that it will be adopted for routine work. Most participants felt that fracture toughness values obtained were technically rigorous for a flat R-curve material in the absence of environmental effects. The extra work entailed in precracking was felt to be worthwhile in terms of the quality of the result. Several labs reported problems with SEPB elevated-temperature testing since precracks are prone to heal.

In overall summary, the round-robin was felt to be a success. Reasonably consistent results were obtained for the IS and SEPB methods between most laboratories for two different materials. Several areas were identified where refinements could be made and there now is greater confidence by the U.S. participants in these two methods. Participants either successfully tried the IS or SEPB methods for the first time, or refined their usual procedures. The IF method was less successful in this round-robin.

A single value of fracture toughness for the silicon nitride seems to be appropriate. No R-curve or environmentally assisted crack growth phenomena were detected. Several questions of interpretation of fracture toughness were raised for the case of the ZAC, which exhibited R-curve and environmentally assisted crack growth. There is no simple interpretation of fracture toughness for this material.

A direct result of this round-robin is that the IS and SEPB methods are now under consideration in ASTM Committees C-28, Advanced Ceramics and E-24, Fracture Testing as candidates for standard test methods for advanced ceramics.

## Figures and Tables

**Table 1:** U.S. participants

Laboratory
NIST
Ceramics Division,
Gaithersburg, MD
Allied-Signal,
Garrett Auxiliary Power Division,
Phoenix, AZ
St. Gobain
Norton Industrial Ceramics Corp.,
Northboro, MA
NASA/Lewis Research Center
Cleveland, OH
Worcester Polytechnic Institute
Department of Mechanical Engineering,
Worcester, MA

**Table 2:** Indentation loads for IF analysis

Material	Load (N)				
silicon nitride	49	98^a^	196^a^	294	
ZAC		98		294^a^	490^a^

a Specified by the IF instructions.

**Table 3:** Indentation strength (IS) specimen initial crack sizes

Material	Indent load *P* (N)	Surface length 2*c* (mm)	Flaw depth *c* (mm)	Depth ratio *c*/*W*
Si_3_N_4_	49	0.165	0.083	0.028
Si_3_N_4_	294	0.570	0.285	0.095
ZAC	98	0.179	0.090	0.030
ZAC	490	0.622	0.311	0.104

**Table 4:** Precrack inspection procedures

Lab	Procedure	
	ZAC	Silicon nitride
NIST	Green felt-tip pen	(Fluorescent dye penetrant-unsuccessful) Low-angle incident lighting
USA lab 2	Commercial blue dye penetrant	Commercial blue dye penetrant
USA lab 3	Felt-tip pen	Blue food coloring, applied under load
USA lab 4	(Red dye penetrant unsuccessful)	(Red dye penetrant unsuccessful)
	Low-angle incident lighting	Low-angle incident lighting
USA lab 5	Acetone and dye,fractographic inspection	Acetone and dye, fractographic inspection

**Table 5:** Indentation fracture sampling. Fraction of indentations that met the *c*/*a* > 2.3 criterion

USA lab	Material			
	ZAC		Silicon nitride	
	294 N	490 N	98 N	196 N
1	0/10	7/10	7/10	10/10
2	20/20	18/20	29/32	29/29
3	5/10	3/10	6/10	10/10
4	10/10	10/10	10/10	10/10
5	0/20	10/20	8/20	14/18

**Table 6:** Summary of fracture toughness values (MN/m1.5)

Method	Silicon nitride	ZAC
IS (low load) (high load)	**5.7**^a^	6.7^b^
	6.3^b^	7.4^b^
SEPB	**5.6**^a^	5.4 (slow rate)
		6.1 (fast rate)
IF	**5.4**^a^ (veiy high scatter)	?

a Denotes the most probable value for *Ku* for silicon nitride.

b No correction for stress gradient.

**Table 7:** Participants utilization of the fracture toughness methods

Laboratory	VAMAS round-robin tests			Other tests
	IS	IF	SEPB	
NASA-Lewis	a	d	a	aCN (Chevron Notch)
Norton	a	b	b	
Allied Signal (Garrett)	d	d	d	aCN (Chevron Notch)
Worcester	b	b	a	
Polytechnic				
NIST	a	a	c	bDCB (Double Cantilever Beam)
				bAM-DCB (Applied Moment DCB)

a Test method already in routine usage, and is preferred.

b Test method already in routine usage.

c Test method will be used.

d Test method not ordinarily used.

## Footnotes

1 NTK Technical Ceramics, NGK Spark Plug Co. Nagoya Japan.

2 Certain commercial equipment, instruments, or materials are identified in this paper to specify adequately the experimental procedure. Such identification does not imply recommendation or endorsement by the National Institute of Standards and Technology, nor does it imply that the materials or equipment identified are necessarily the best available for the purpose.

3 Instron model TTCML with a 50000 N load cell.

4 Instron model 1122 with a 5000 N load cell.

5 This stress intensity shape factor variability is a serious interfering factor on measurements of R-curves in IS testing. Krause [26] and Anderson and Braun [28] have also made this observation.

6 Stable crack extension is possible in flat *R*-curve materials for the precrack sizes specified in the VAMAS round-robin, but only if the testing system is extremely rigid. Baratta and Dunlay [38] and Sakai and Inagaki [39] have defined the geometries, specimen and system compliances that lead to stable crack extension. The testing systems used in the present round-robin were usually “soft” or compliant, and would not promote stable crack growth.

7 In defense of the choice of three-point loading, it should be stated that the scheme was presumably chosen to be in compliance with the loading configurations of ASTM standard E-399 [11]. The latter standard uses much larger specimen sizes that are easier to set-up however.

8 (0.9)^−1.5^ = 1.17; to (1.1) ^−1.5^ =0.87.
